# Nonrandomised controlled trial in recurrent glioblastoma patients: the promise of autologous tumour lysate-loaded dendritic cell vaccination

**DOI:** 10.1038/s41416-023-02194-1

**Published:** 2023-02-15

**Authors:** Francesco Pasqualetti, Sofia Zanotti

**Affiliations:** 1 Department of Oncology, University of Oxford, OX3 7DQ Oxford, UK; 2 Radiation Oncology, Azienda Ospedaliero Universitaria Pisana, Pisa, Italy; 3 Anatomic Pathology Unit, IRCCS Humanitas University Research Hospital, Milan, Italy

**Keywords:** CNS cancer, Cancer immunotherapy

## Abstract

Recently, Liau et al. reported the results of Phase 3 clinical trial testing DCVax-L vaccines on patients with glioblastoma. Despite the promising and significant results obtained, the study design and the long-lasting period of recruitment of this work deserve some reflection.

Glioblastoma (GB) is a malignant tumour that is thought to arise from neural stem cells or progenitor cells residing in the central nervous system. About 90% of the GB cases are diagnosed as primary GB and undergo intensive multi-modal therapy [1]. However, despite the advances recorded in many other solid cancers, the success rate of current treatments for GB is disappointingly low and survivors suffer from multiple therapy-related long-term side effects, warranting the urgent need for more effective therapies [1–3]. After decades of intensive research, most attempts to further improve GB therapies within randomised trials failed to deliver significant results. The success rate of current agents tested in combination with radiotherapy remains disappointingly low as there is no additional advantage in terms of overall survival (OS).

Immunotherapies have long been considered a promising form of cancer treatment that exploit the patient’s immune response against tumours [4]. In essence, immunotherapy compounds act by interfering with immune checkpoint blockade agents, but can also be based on monoclonal antibodies or therapeutic vaccines [5]. Moreover, the stimulation of the immune system was shown to work synergistically with checkpoint inhibitors by producing a strong antitumor immune response [6]. The lysate-loaded dendritic cell (DCVax-L) vaccine is a cell-based vaccine able to stimulate a polyclonal T-cell response by triggering the antigenic repertoire of the patient’s own dendritic cells. Recently, Adam and colleagues discussed the efficacy of DCVax-L vaccines in combination with other standard systemic therapies in the melanoma field, a characteristically immunogenic cancer [7]. Their work suggests that the DCVax-L vaccine is well tolerated and can be adopted as a treatment option in patients with recurrent and metastatic melanoma [7]. Although the DCVax-L vaccine can trigger the patient’s immune response, specifically T cells, it remains a therapy that has to be accompanied by additional therapeutic agents and has thus found minor success as monotherapy.

Recently, Liau et al. reported the results of Phase 3 clinical trial testing DCVax-L vaccines on patients with either newly diagnosed or recurrent GB. The trial has called attention to the beneficial relevance of DCVax-L vaccines by observing a significant improvement in OS of GB patients [8]. This data could be suggesting that the combination of the DCVax-L vaccine and standard of care may offer the ability to initially cause a T-cell response and further protect the T cells from immunosuppression. To overcome the limits related to the use of a cross-over design in assessing the impact of DCVax-L in OS, an external control group composed by patients treated with standard of care (radiotherapy with TMZ) within selected randomised trials were included.

The authors showed promising results of the DCVax-L vaccine in respect to the external cohort of patients. The median survival from the date of randomisation of the patients treated with the DCVax-L vaccine was 19.3 months (95% CI, 17.5–21.3) whereas for the 1366 patients in the control group was 16.5 months (95% CI, 16.0–17.5) (log-rank HR, 0.80; 95% CI, 0.00–0.94; *P* = 0.002). Survival at 48 months after randomisation was reported to be 15.5% and 9.9% in the treated and control arms, respectively. Despite the promising and significant results obtained, the study design and the long-lasting period of recruitment of this work deserve some reflection.

The studies that analysed a selected control group showed a significant overlap of clinical characteristics and enrolment period when compared to the work performed by Liau and colleagues (from 2007 to 2015). However, the choice of patients enrolled in the control group from different clinical trials cannot entirely eliminate the limitations encountered when adopting a cross-over design, even if matched by several criteria [9]. Upon further evaluation of the studies selected by Liau et al. to assess the impact of the vaccine in patients with newly diagnosed GB, the authors reported incomplete demographic and clinical characteristics. For example, the information about the patient’s age was missing in 2/5 trials and the assessment of the extent of resection, thus an indicator for the persistence of residual disease was not included for 3/5 trials. Similarly, three of the comparison studies on the recurrence of GB did not provide data on age while for five studies the definition of *MGMT* was absent. Furthermore, *IDH* mutations were not evaluated in any of the selected studies. Yet, the authors performed an accurate randomised part of the study as, in addition to standard radio-chemotherapy, 99 patients received a placebo whereas 232 the active treatment. Nevertheless, the results of this randomisation should be further analysed by intention to treat [10].

Moreover, the long duration of the enrolment period indicates that the criteria adopted for recruiting GB patients do not consider the latest WHO classifications [1]. Consequently, this suggests that the patients alive at 48 months might have less aggressive tumours, thus an *IDH* 1 and 2 mutated glioma. Finally, although it is accepted that the pseudo-progression might be an issue when assessing an actual disease recurrence, analysis of progression-free survival did not show significant differences between immunotherapy-treated patients and controls.

To demonstrate that the efficacy of the new DCVax-L-based therapy provides additional benefits in comparison to the standard of care, the results reported by Liau et al. should be confirmed by a randomised trial. Further studies should also evaluate the effect of the DCVax-L vaccine in combination with other standard systemic therapies routinely used for the treatment of GB. Considering the challenges encountered during the planning of clinical studies for newly diagnosed GB without accounting for the cross-over effect after progression, recurrent GB that were not previously treated with the DCVax-L vaccine could be regarded as the most suitable setting for planning prospective randomised trials. Appropriate novel trial designs, for example, window trials, could be considered.

An important underlying theme to many of the recommendations made is that trial-based studies should ensure standardisation of data acquisition and analysis. This in turn, may be exploited to valorise the potential of DCVax-L vaccine-based therapies which are gradually being unveiled and could provide guidance towards additional therapy options in the GB field.

## Data Availability

Not applicable.
